# Indian research on women and psychiatry

**DOI:** 10.4103/0019-5545.69237

**Published:** 2010-01

**Authors:** Rakesh K. Chadda, Mamta Sood

**Affiliations:** Department of Psychiatry, All India Institute of Medical Sciences, New Delhi - 110 029, India

**Keywords:** Women and Psychiatry, reproductive phases and mental health, family planning and mental health

## Abstract

The paper discusses research on various issues related to mental health specific to women, published mainly in the Indian Journal of Psychiatry. We carried out a manual search of all the issues of the journal. Indian psychiatrists have worked in a wide range of areas including psychological aspects of different reproductive phases like pregnancy, puerperium, menopause, menstrual cycle, psychological consequences of contraception, infertility and surgical loss of uterus or breast. Most of the studies are cross sectional with very few prospective studies. There is a need for longitudinal, epidemiological and intervention studies.

## INTRODUCTION

Gender has been described as a critical determinant of mental health and mental illness.[1] The specialty of gynaecology and obstetrics in medical science exclusively caters to the specific health needs of the women, whereas there is no such exclusive medical discipline for men. The reproductive system of women also follows a unique biological rhythm and their body undergoes specific changes with different cycles of reproduction, both anatomical as well as physiological. Menarche, puberty, menstrual cycle, pregnancy, puerperium and menopause are specific life events of a woman’s life. These phases are associated with different kinds of stress; and if a woman is not able to cope with the changes or if the social support systems fail, she may develop mental health problems.

Gender differences in mental disorders have been reported, particularly, in the prevalence of common mental disorders including depression, anxiety disorders and somatoform disorders.[2,3] Depression is not only the most common women’s mental health problem but may be more persistent in women than men. However, alcohol dependence, another common disorder, is twice more common in men than in women. Men are also three times more likely to be diagnosed with antisocial personality disorder than women. There are no marked gender differences in the rates of severe mental disorders like schizophrenia and bipolar disorder that affect less than 2% of the population, though some differences have been reported in age of onset of symptoms, frequency of psychotic symptoms, course of these disorders, social adjustment and long term outcome. Thara and Rajkumar[4] have reported better course and outcome in women in schizophrenia as compared to men.

Higher rates of depression, anxiety and somatic symptoms are related to a range of risk factors such as gender-based roles, stressors and negative life experiences and events. Gender specific risk factors for common mental disorders that disproportionately affect women include gender-based violence, socioeconomic disadvantage, low income and income inequality, low or subordinate social status and rank, and unremitting responsibility for the care of others.[3,5] There is a positive relationship between the frequency and severity of such social factors, and the frequency and severity of mental health problems in women. Severe life events that cause a sense of loss, inferiority, humiliation or entrapment can predict depression. Women are often exposed to sexual violence which leads to high rates of post traumatic stress disorder (PTSD) following such violence.

Psychiatric conditions seen exclusively in women include those seen in association with various phases of the sexual maturation or reproductive phases like pregnancy, menstrual cycle, puerperium, breast feeding, mothering and menopause, and surgical procedures related to specific female organs like mastectomy and hysterectomy.[1]

This paper discusses research on psychiatric problems seen in women, published mainly in the Indian Journal of Psychiatry. A manual search of all the volumes of the Indian Journal of Psychiatry was carried out. We were able to identify two presidential addresses[6,7] one editorial,[8] 19 original papers, four case reports and one oration,[9] which have discussed exclusively women related mental health issues.

Indian psychiatrists have studied psychiatric morbidity in women in primary care settings, mental health abnormalities associated with various phases of reproductive cycle like menstrual phases, pregnancy and postpartum period, psychiatric aspects of contraception and medical termination of pregnancy, infertility and hysterectomy, and teratogenecity. Important research findings are discussed below.

### Psychiatric morbidity in women in primary care

In a recent study on psychological symptoms in rural women from Tamil Nadu, Lawson *et al*.[10] reported an association between caseness on self report questionnaire (SRQ) and poverty. Domestic violence, alcoholism in husbands, physical illness, pain symptoms, increasing age and having more than two children increased the probability of caseness. Suicidal ideation was reported by nearly half of the sample. Increasing age, being a widow, higher number of children and physical illness in a family member were important contributory factors. However, the study had a major limitation of not including psychiatric assessment in the design.

### Mental health problems associated with different reproductive phases in women

#### Menstrual cycle and mental health

Many Indian psychiatrists have attempted to explore the emotional changes and disturbances in relation with different phases of menstrual cycle;[9] the pre menstrual phase has been found to be associated with emotional distress. In an ICMR supported study on suicide attempters, Prof A Venkoba Rao and Prof S Parvathi Devi in 1972[11] found that 64% of the attempters had made attempts during the pre menstrual phase or early menstrual phase. Marital status was not found to be an influencing factor. The authors explained the findings in term of pre menstrual depression and psycho endocrine imbalance during the period.

Professor Abraham Varghese way back in 1963[12] reported a case of pre menstrual psychosis, a rare disorder, in a 26-year-old lady, in whom psychotic symptoms would appear every time during the pre menstrual phase and would be relieved completely with the onset of menstrual bleeding. The symptoms sometimes would last for periods up to 2 weeks, but were not long standing. At times she had required hospitalization in a state mental hospital and was given ECT for the symptoms. Shah *et al*.[13] reported a case of menstrual psychosis, in which the psychotic disturbance had onset in the pre-menstrual phase and would continue for two to three weeks. Later investigations revealed low progesterone levels in the follicular phase of the menstrual cycles.

#### Pregnancy and post partum period

Pregnancy and post partum period have also attracted the attention of many psychiatrists. There have been both prospective as well as cross sectional studies. In a cross sectional study on phenomenology of post partum psychiatric phenomena by Gautam *et al*.[14] In early 80s from Jaipur, 67% of the patients seen in a psychiatric setting were found to suffer from schizophrenia and 25% from affective psychosis as per ICD 9 criteria, implying that only patients with severe disturbance are likely to reach the psychiatric settings. Sixty six per cent of the cases were primipara.

In a prospective study conducted more than 30 years ago at Vellore, John *et al*.[10] followed up 59 women from the last trimester to the post partum period. Twenty four per cent of them developed depression requiring intervention. Women having higher neuroticism score and those who were more particular about the sex of the baby were more likely to develop depression. Age, parity, family structure, complications during pregnancy and sex of child were not found to have any relationship with tendency to develop depression. In another prospective study on depression during late pregnancy and post partum period conducted in an army hospital setting by Sood and Sood,[16] about 10 years ago, prevalence of depression as measured using the Beck Depression Inventory was 8.3%, 20.0% and 12.8% during the third trimester, three days after delivery and eight weeks after delivery respectively. Incidence was 16% and 10% in early and late post partum periods respectively. Depression during late pregnancy was found to be associated with depression occurring in early post partum period, but not with the late post partum period.

#### Menopause

Women in menopause have been found to suffer higher psychological morbidity as compared to pre menopausal and post menopausal women.The predominant psycho pathology is from depressive spectrum.[17] Various menopausal symptoms reported in Indian settings include physical or mental exhaustion, irritability, depressed mood, decreased sleep and decreased interest in sex.[17,18]

### Family planning and mental health

Increasing population of India has been a major concern with the Government and the health planners, which led to the launch of family planning program in 1960s. Various activities undertaken in the family planning program have been associated with mental health consequences, which have also been the focus of mental health professionals. Mental health aspects of family planning were the topic of an editorial in 1981 by late Professor B.B. Sethi.[8]

#### Contraception and medical termination of pregnancy (MTP)

Tubectomy, one of the common methods of contraception, has been reported to be followed by three sets of symptoms: menstrual, psychological and sexual.[19] There is a wide variation in the frequency of the latter two categories of symptoms, varying from 1.5-83%. Anxiety, depression, memory impairment and somatic complaints are common psychological sequelae.[8,19] Psychological symptoms are less frequent after MTP as compared to those seen after tubectomy or vasectomy. Intrauterine contraceptive devices, however, have not been found to be associated with psychological symptoms.[20] In a study of rejecters and acceptors of MTP amongst women with psychiatric problems, who were advised termination of pregnancy on medical grounds during the first trimester, acceptors were from higher education and socioeconomic background. Age, marital status, number of children, and nature of illness were not found to influence the decision to consent for MTP. Outcome of psychiatric illness was found to be better in the acceptors of MTP than the rejecters.[21]

#### Infertility

Psychological consequences of infertility have also been investigated. In a controlled study, Thara *et al*.[22] found that couples suffering from infertility suffer more psychological symptoms like depression and anxiety and also psychosexual dysfunction. The males suffer erectile dysfunction and premature ejaculation, whereas the females suffer vaginismus, dyspaerunia and sexual dysfunction.

### Surgeries related to women specific organs

Uterus and breast are two unique organs of women, which are associated with female image and are also vital for reproduction and mothering. Both the organs are prone to cancer and their loss is associated with psychological and social consequences as well as important physical implications because of the seriousness of the illness and importance of the two organs in a woman’s body image, sexuality and motherhood. There have been a few studies on the topic in India.

In a prospective study of psychiatric complications of hysterectomy, Subramaniam *et al*.[23] conducted a one-year follow up of 50 women who had undergone hysterectomy. Twenty per cent of the patients developed psychiatric problems, mainly depression, Whereas only one of the 20 controls developed hysterical reaction. Patients who had developed depression had higher scores on neuroticism even before hysterectomy than those who did not develop any psychiatric problem. Patients who had undergone hysterectomy reluctantly had higher risk of developing psychiatric symptoms than those who had undergone surgery willingly. The authors concluded that patients undergoing hysterectomy need to be prepared properly and counseled so as to prevent psychiatric complications.

Mastectomy is the other major surgery, which women may have to undergo. Following mastectomy, a woman may experience a range of concerns and fears related to physical appearance and disfigurement, uncertainty about recurrence and the fear of death. Mahapatro and Parkar[24] studied psychological consequences in women who had undergone mastectomy or lumpectomy for breast cancer. Mastectomy is a more radical procedure than lumpectomy. They found concerns with body image or disfigurement only in mastectomized group. The concerns about the illness and after effects were generally resolved in both the groups except for sexual role and performance, which were resolved to a lesser extent in the mastectomized group. The two groups suffered similar levels of anxiety and depression. The authors concluded that the concern regarding sexual role and performance are resolved to a lesser extent in the mastectomized group and specific psychological intervention is required for them to enhance their coping strategies with regard to concerns of body image, and sexual role and performance

## CONCLUSION

Indian psychiatrists have investigated a wide range of mental health problems in women including those occurring during pregnancy and puerperium, psychological effects of contraception, MTP, hysterectomy and mastectomy, suicide, relationship between domestic violence and mental health, suicidal behavior and epidemiological trends. Prospective and intervention studies are generally lacking.
